# Supplementary material for: Single-cell analysis reveals diversity of tumor-associated macrophages and their interactions with T lymphocytes in glioblastoma
Source: Sci Rep. 2023 Nov 27;13:20874. doi: 10.1038/s41598-023-48116-2 (PMC10682178; doi:10.1038/s41598-023-48116-2)
Supplement: Supplementary file 1 — Supplementary Information 1. [file 41598_2023_48116_MOESM1_ESM.pdf]

Supplementary Figure 1
Stacked bar plot displaying frequency of all cell types in GBM samples from dataset used.
