# Supplementary figures and images for: Single-cell analysis reveals diversity of tumor-associated macrophages and their interactions with T lymphocytes in glioblastoma
Source: Sci Rep. 2023 Nov 27;13:20874. doi: 10.1038/s41598-023-48116-2 (PMC10682178; doi:10.1038/s41598-023-48116-2)

## All cells

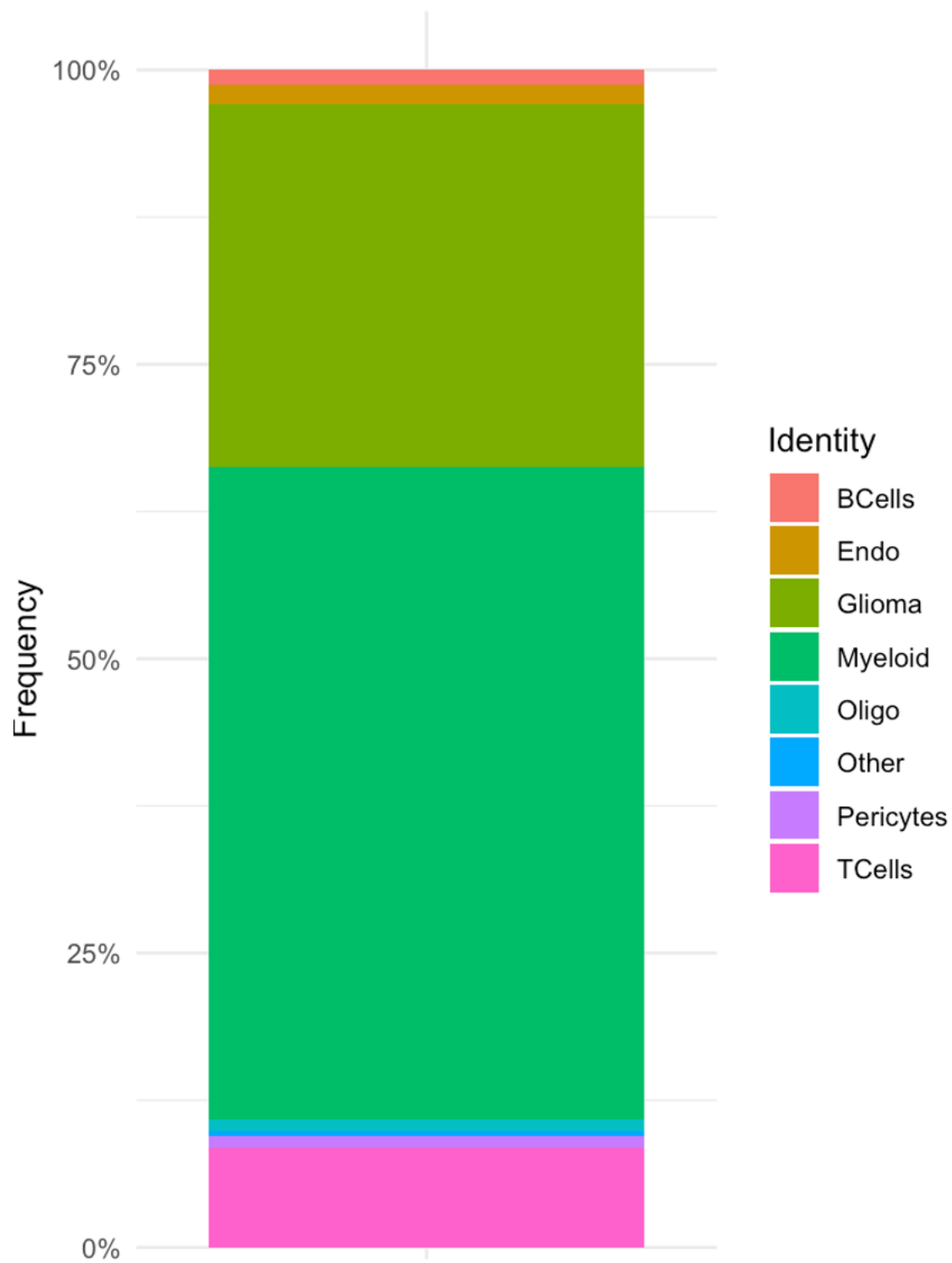

Supplement: Supplementary file 2 — Supplementary Information 2. [file 41598_2023_48116_MOESM2_ESM.pdf]
